# Drosophila C Virus and La Jolla Virus Formulations for Plant Protection Against Spotted-Wing *Drosophila*

**DOI:** 10.3390/insects16121258

**Published:** 2025-12-11

**Authors:** Monja Jochmann, Sven Sölmann, Thorsten Gröb, Martin Wortmann, Kwang-Zin Lee, Michael W. Wolff, Waldemar Keil, Anant V. Patel

**Affiliations:** 1 Faculty of Engineering and Mathematics, Fermentation and Formulation of Biologicals and Chemicals, Hochschule Bielefeld—University of Applied Sciences and Arts, Interaktion 1, 33619 Bielefeld, Germanyanant.patel@hsbi.de (A.V.P.); 2 Department of Technology, Bielefeld University, Universitätsstr. 25, 33615 Bielefeld, Germany; 3 Institute of Bioprocess Engineering and Pharmaceutical Technology, University of Applied Sciences Mittelhessen (THM), 35390 Giessen, Germany; thorsten.groeb@lse.thm.de (T.G.); michael.wolff@lse.thm.de (M.W.W.); 4 Faculty of Physics, Bielefeld University, Universitätsstr. 25, 33615 Bielefeld, Germany; 5 Fraunhofer Institute for Molecular Biology and Applied Ecology, Branch of Bioresources, Ohlebergsweg 12, 35392 Giessen, Germany; kwang-zin.lee@ime.fraunhofer.de

**Keywords:** entomopathogenic viruses, formulation, chitosan, biocontrol, fruit pests, encapsulation

## Abstract

The biological control of *SWD* using entomopathogenic viruses offers a sustainable alternative to chemical insecticides; however, viral instability under environmental and digestive conditions limits their application. Here, we present a chitosan–TPP encapsulation system designed to protect and control the release of Drosophila C virus (DCV) and La Jolla virus (LJV). Physicochemical characterization by dynamic light scattering (DLS) and zeta potential measurements confirmed successful encapsulation and indicated that stable electrostatic complexes form within a narrow pH range where chitosan is positively charged and the virion surface is negatively charged. The encapsulated viruses remained intact under acidic conditions but were rapidly released under alkaline conditions mimicking the posterior midgut of *SWD*. Encapsulation efficiency increased with decreasing chitosan molecular weight and higher stirring speed, reaching up to 90%. Bioassays demonstrated that both formulated viruses caused high mortality in adult flies, with no adverse effects from the carrier matrix. These findings establish chitosan–TPP formulations as an effective strategy for virus stabilization and targeted release in biological pest control.

## Simple Summary

The Spotted-Wing *Drosophila* (*SWD*) is an invasive pest that causes major losses in fruit production worldwide. Biological control using naturally occurring viruses provides an environmentally friendly alternative to chemical pesticides but is limited by viral instability under field and digestive conditions. In this study, we developed a protective formulation using chitosan, a natural biopolymer, and the crosslinker tripolyphosphate (TPP). The chitosan–TPP coating forms a thin layer around virus particles, shielding them from acidic degradation in the fly’s gut and releasing them only in the posterior midgut—the site of infection. Assays showed that the encapsulated viruses remained stable, were efficiently released under alkaline conditions and significantly reduced fly survival, while the coating itself had no harmful effects. This chitosan-based encapsulation approach offers a promising, sustainable strategy for viral pest control, with potential to lower pesticide use and protect beneficial insects and the environment.

## 1. Introduction

Viruses replicate by infecting host cells, leading to outcomes that range from mild illness to severe disease and even death [1,2]. Due to their role as pathogens, viruses are often perceived negatively, which is understandable given the millions of deaths they cause annually [3]. Nonetheless, viruses are attractive candidates for biological crop protection because of their high host specificity, which minimizes risks to non-target organisms and the wider environment. Given that viruses occur across nearly all life forms—there is theoretically a virus for every major pest species [4,5,6]. Nevertheless, viruses also hold significant potential in both medicine and agriculture. In medicine, they can be harnessed for diverse applications, including vaccine development, targeted cancer therapies [7,8] and gene therapy [9,10]. In agriculture, viruses have been investigated as biological control agents against insect pests, plant pathogens and other agricultural threats [11,12,13]. Despite this potential, the practical use of viruses in agriculture remains limited [14]. One key challenge is that only about half of the active virus formulations produced are utilized, largely because of difficulties in handling and storage. In particular, maintaining a cold chain for field applications is often impractical and prohibitively costly [15].

One invasive pest of major concern is *Drosophila suzukii*, Spotted Wing *Drosophila* (*SWD*). First observed in Japan in 1916 [16], it has spread rapidly globally since the late 2000s [17,18,19,20]. Unlike most *Drosophila* species, *SWD* can puncture the skin of intact, ripening fruit to lay eggs beneath the surface [21,22,23,24]. Infestation renders fruit unmarketable, leading to substantial economic losses [21,25,26]. Because the eggs and larvae develop deep within fruit tissue, they are largely unaffected by conventional surface-applied insecticides [27]. Potential control of adults coincides with fruit ripening and harvest, which restricts the use of broad-spectrum insecticides due to consumer safety concerns [28].

**Figure 1:**
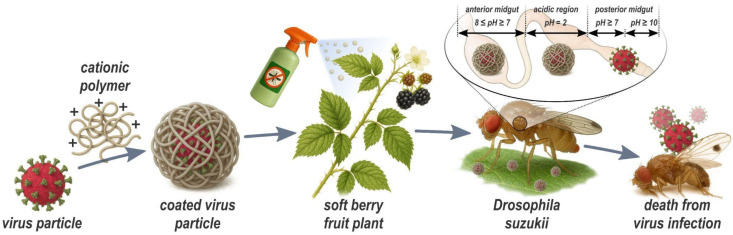
Overview of production and mode of action of virus-containing spray formulations developed in this study. The Drosophila C Virus or La Jolla Virus is encapsulated, employing low molecular weight chitosan and embedded in a formulation that is sprayable. *Drosophila suzukii* is attracted and ingests the coated virus through its proboscis. In the anterior midgut and in the acidic area, the virus particles are protected from enzymatic and chemical degradation by the coating. In the posterior midgut (site of viral infection) the virus is released by an alkaline pH-shift and absorbed by the intestinal cells. The scheme is for illustrative purposes only.

Viruses represent promising biological alternatives for pest management, as they can be transmitted both horizontally and vertically, potentially enabling more effective long-term control of *SWD* [29]. Since the 1970s, baculoviruses have been used as biopesticides against the codling moth, and research in this area has largely focused on formulation additives, given that baculoviruses are enveloped and therefore inherently more stable [14,30,31]. The viral envelope provides protection against the host immune system by reducing the likelihood of immune recognition. In addition, the envelope contributes to the stability of the virion by offering increased resistance to UV and temperature fluctuations, thereby enhancing the virus’s overall shelf life [15,30,32]. Several viruses lethal to *SWD* have been identified, including Drosophila C virus (DCV), Cricket Paralysis virus (CrPV), and Flock House virus (FHV). In addition, natural viruses such as Drosophila A virus (DAV) and La Jolla virus (LJV) have been described. Intrathoracic injections of these viruses have shown high lethality in *SWD*. However, promising candidates like DCV and LJV are non-enveloped and thus considerably less stable under environmental conditions [33,34]. For successful field application, these viruses require protective formulations that shield them from environmental stressors. Furthermore, injection is impractical for field applications [35,36,37,38]. For high efficacy, viral pest-control strategies require formulations that (i) maintain virulence under environmental stress (biotic and abiotic) and (ii) enable oral delivery by ensuring viral particles remain intact through the insect’s gut until released in the posterior midgut. The main aim of this study is to formulate the DCV and LJV viruses in order to protect them from biotic and abiotic stress and obtain a pH induced release in the posterior midgut of *SWD*. Therefore, we investigated the DCV and LJV viruses for their potential control of *SWD*. DCV was propagated in vitro employing Schneider (S2) cells, purified, and formulated into chitosan–tripolyphosphate (TPP) polyelectrolyte complexes, yielding nanocapsules optimized for ingestion. In parallel, we developed an analogous nanocapsule of LJV. To our knowledge, this is the first demonstration that cell-culture-derived LJV exhibits lethal effects on *SWD*, highlighting its promise as a novel biocontrol agent. Together, these formulations provide evidence of stabilizing the viruses from biotic and abiotic stress. Overall, we derive a concept, depicted in Figure 1 for virus-based biological protection against *SWD*.

Future field trials will be essential to assess the effectiveness of these virus formulations under realistic environmental conditions; however, current regulatory constraints [39] on the release of viral biocontrol agents pose substantial challenges for conducting such studies.

## 2. Materials and Methods

*Drosophila melanogaster* Schneider 2 (S2) cells (Deutsche Sammlung von Mikroorganismen und Zellkulturen, ACC 130, Braunschweig, Germany) were used for the in vitro production of DCV and LJV. Cells were maintained under sterile conditions in Sf-900™ II SFM insect cell medium (Gibco, Grand Island, NY, USA). Suspension cultures were grown in 100 mL baffled shake flasks (Carl Roth GmbH + Co. KG, Karlsruhe, Germany) with a working volume of 20 mL at 28 °C and 85 rpm. Cells were subcultured twice weekly when the viable cell density reached 1–2 × 10^7^ cells mL^−1^. For new cultures, cells were inoculated at 1–1.5 × 10^6^ cells mL^−1^. Cell density and viability (≥95%) were determined using a Guava^®^ EasyCyte 5HT flow cytometer (Luminex Corp., Austin, TX, USA) or by manual counting with a hemacytometer and propidium iodide or trypan blue exclusion. A DCV stock solution (7 × 10^10^ genome equivalents mL^−1^) was used as inoculum. S2 cells in exponential growth were adjusted to 1–2 × 10^6^ cells mL^−1^ and pre-incubated for 24 h prior to infection.

For DCV a MOI of 10 was chosen, For LJV a MOI of 1. The required inoculum volume (V_virus_) was calculated according to:

(1) $$V_{virus}=\frac{V_{cells}\cdot c_{cells}\cdot MOI}{c_{virus}}$$

where V_cells_ is the total culture volume, c_cells_ the cell density at infection, and c_virus_ the titer of the virus stock. The calculated virus volume was added to the S2 culture and gently mixed. Cultures were incubated post-infection at 28 °C and 85 rpm. The time of harvest (TOH) for DCV was 1–3 days post-infection. TOH of LJV was 3–4 days post infection. Cultures were frozen at −80 °C overnight to lyse cells and release intracellular virions. After thawing, suspensions were vortexed for 2 min and clarified by centrifugation at 4 °C for 15 min (4650× *g* for 50 mL tubes, or up to 16,000× *g* for smaller volumes). The supernatant, containing crude DCV/LJV, was transferred to sterile tubes and subjected to a depth filtration (0.45 µm; Sartorius Stedim Biotech GmbH, Göttingen, Germany) to remove remaining cells and cell debris. The subsequent chromatographic purification was performed by a membrane-based steric exclusion chromatography (SXC) using 8% polyethylene glycol (PEG) 8000 (Carl Roth GmbH + Co. KG, Karlsruhe, Germany) and regenerated cellulose membranes with a nominal pore size of 1 μm (Whatman RC60, GE Healthcare Life Sciences, Düsseldorf, Germany) at pH 7.4, as previously described [40,41,42].

Viral RNA was quantified by Reverse Transcription Quantitative Polymerase Chain Reaction (RT-qPCR) on a C1000 TouchTM Thermal Cycler (Bio-Rad, Hercules, CA, USA) using the DCV-specific primers DCV-Forward (5′-TCATCGGTATGCACATTGCT-3′) and DCV-Reverse (5′-CAGAAGAGCATGGTTATGCG-3′) and the SensiFAST™ SYBR^®^No-ROX One-StepKit (Meridian Bioscience, Cincinnati, OH, USA). A standard curve was prepared with a 10-fold dilution series of a viral standard containing a genomic titer from 10^11^ GE/mL to 10^2^ GE/mL. Reverse transcription was performed at 45 °C for 10 min. After initiating reaction at 95 °C for 2 min, 40 cycles of denaturation (15 s, 95 °C), primer hybridization (55 s, 55 °C) and elongation (10 s, 68 °C) followed.

Chitosan (0.5%) in 1% acetic acid (*w*:*v*), TPP (0.1%) in MilliQ (*w*:*v*) and DCV/LJV with a final titer of 5 × 10^8^ GE/mL in PBS (pH 5) were used in a ratio of 1:1:2. Chitosan solution and DCV are added together. TPP is added dropwise with vigorous mixing (e.g., 16,000 rpm) for 30 s. The particles are separated from the reaction solution by centrifugation (2500× *g*, 5 min) and resuspended in PBS (pH 5). Four different chitosan solutions with varying molecular weights were used, high molecular weight chitosan (350 kDa), low molecular weight chitosan (50 kDa), very low molecular weight chitosan (20 kDa) ultra-low molecular weight chitosan (15 kDa). The encapsulation efficiency (EE) was determined by calculating the ratio between the viral genomes in the capsules and the non-encapsulated copies of the viral genomes:

(2) $$EE\%=\frac{{Input}_{virus}-{Supernatant}_{virus}}{{Input}_{virus}}\;\cdot 100\%$$

The microparticles were separated by centrifugation (2500× *g*, 5 min) and later dissolved by increasing the pH to a value above pH 10 using NaOH (2 mol/L). The RNA from the released viruses of the dissolved capsules and the viruses from the supernatant was separately isolated using the Quick-RNA™ Viral Kit (Zymo Research, Irvine, CA, USA).

The hydrodynamic diameter (Z-Average) of the samples was determined employing dynamic light scattering (DLS, Delsa Nano C, Beckman Coulter GmbH, Aachen, Germany) with a scattering angle of 165°. Approximately 80–100 µL of each sample was added to disposable UV micro cuvettes (Z-height 8.5 mm, 70–850 µL, Brand GmbH und Co. KG, Wertheim, Germany) and placed into the cell counting chamber. If necessary, high-concentration colloidal suspensions were diluted with MilliQ water to ensure accurate measurements. 70 scans were recorded to calculate the number, volume, and intensity distributions, as well as the Z-average (or cumulant result) according to ISO standards, along with the polydispersity index (PDI). Parameters used for measurements can be found in the Supplementary Information. Zeta potentials (ζ) were determined using electrophoretic light scattering with the same Delsa Nano C instrument. To ensure the absence of air bubbles in the measurement flow cell, 800–1200 µL of sample volume were injected into the flow cell using sterile syringes (Thermo Fisher Scientific, Waltham, MA, USA). The formulated and unformulated virus particles were imaged using a scanning electron microscope (Carl Zeiss AG, Jena, Germany) with an acceleration voltage of 10 keV. The formulated virus particles are prepared and subsequently divided into separate reaction vessels that are available for each desired time interval. The pH value of the reaction vessels is adjusted using NaOH (2 mol/L). After varying time intervals (1–30 min), the samples are centrifuged for one minute at a speed of 15,000× *g*. The supernatant is separated and the RNA of the released viruses is isolated and quantified using RT-qPCR. 20 female flies (3–7 days old) were fed LJV/DCV suspensions in 100 mM sucrose in PBS (pH = 5) at concentrations of 5 × 10^8^ GE/mL in a standard 2.5 cm vial containing a piece of folded paper towel. The negative control was 100 mM sucrose in PBS (pH = 5) alone. The flies were maintained as described above and were provided with 100 µL 100 mM sucrose daily. Survivors were counted daily. The experiments featured three biological replicates, each consisting of three technical replicates. The results are presented as means of all nine replicates.

## 3. Results

### 3.1. Virus Formulation

**Figure 2:**
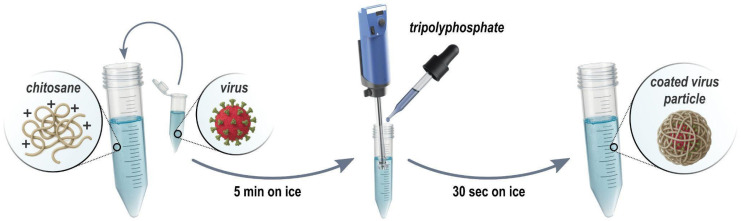
The formulation process of chitosan–Tripolyphosphate coating. First, chitosan is mixed with the Drosophila C/La Jolla virions. Subsequently, Tripolyphosphate is added dropwise under rigorous (Ultraturrax) stirring resulting in coated virus particles.

From a wide array of formulation agents using chitosan for diverse applications, our focus now shifts to the formulation of DCV and LJV virions utilizing chitosan and Tripolyphosphate (TPP). This formulation is specifically designed to meet two essential protective requirements: (i) ensuring the virus is shielded from denaturation caused by harsh conditions in the open field such as high temperatures. Furthermore, providing additional protection against (ii) enzymatic degradation in the anterior midgut of *SWD*. Here, a highly acidic environment (pH ≈ 2) keeps the formulation stable until the virus is released by more alkaline conditions of the posterior midgut, where it enters epithelial cells. To achieve these aims, we have chosen chitosan, a well-abundant cationic biopolymer, in combination with TPP, which acts as a cross-linking agent to enhance the stability and protective capabilities of the virus formulation. This approach relies on the electrostatic interactions between chitosan TPP and the virion surface to create a formulation that offers effective protection and controlled release of the virus under the desired basic conditions. The hypothesis is that the release of virions from the formulation occurs upon deprotonation of chitosan amine groups in basic pH but is prevented in acidic pH. To monitor the formation of particles during the formulation (Figure 2) of the DCV or LJV virions with chitosan and TPP, DLS measurements were performed on the pristine virions and the final formulations.

### 3.2. Physicochemical Characterization of Virus Formulations

**Figure 3:**
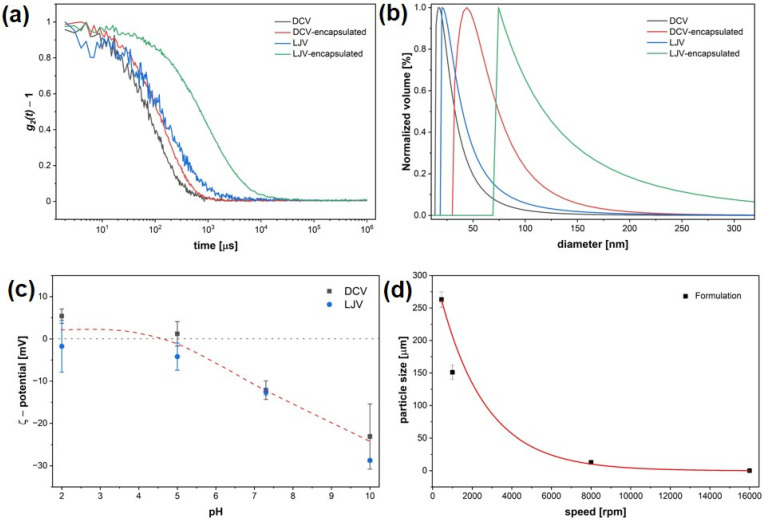
Physicochemical characterization of Drosophila C Virus and La Jolla Virsu formulations. (**a**) Dynamic Light Scattering autocorrelation functions of free and encapsulated virions. (**b**) Size distributions of hydrodynamic diameters before and after encapsulation. (**c**) Zeta potential of Drosophila C Virus and La Jolla Virus across a pH range, with the isoelectric point close to pH 5. (**d**) Particle size as a function of stirring speed.

DLS measurements confirmed successful encapsulation of DCV and LJV in chitosan–TPP complexes. The autocorrelation functions (Figure 3a) revealed a clear shift to larger correlation times for both encapsulated virions compared to the free virions.

Correspondingly, volume distribution analysis of the hydrodynamic diameter (Figure 3b) indicated that both DCV- and LJV-containing formulations exhibited increased hydrodynamic diameters compared to the non-formulated viruses. The ζ potential of DCV and LJV was examined using ELS across a range of different pH values (Figure 3c). Both DCV and LJV displayed a gradual decrease in surface potential with increasing pH, confirming the expected transition from near-neutral to negatively charged particles. Based on these results, only a narrow pH window around pH 6 exists, in which both virions carry a negative surface charge while chitosan remains positively charged.

Furthermore, the relationship between stirring speed during formulation and the resulting particle size followed an exponential decay. Increasing the stirring speed from low to high rpm led to a substantial reduction in particle diameter—from approximately 250 μm at low speed to below 100 nm at the highest speed tested (16,000 rpm), indicating that more intense mixing conditions promote the formation of smaller particles.

### 3.3. Morphology

**Figure 4:**
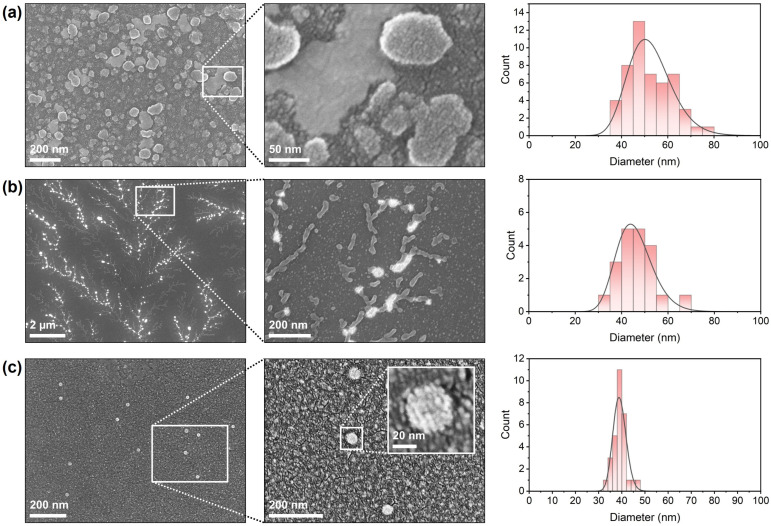
SEM images and resulting size distributions of (**a**) formulated Drosophila C Virus, (**b**) untreated Drosophila C Virus and (**c**) untreated La Jolla Virus.

SEM was employed to visualize the morphology and surface characteristics of the formulated and untreated viral samples, as well as to determine particle size distributions (Figure 4). The formulated DCV sample (Figure 4a) exhibited a dense population of nano-sized, spherical particles distributed uniformly across the substrate surface. At higher magnification, individual particles showed smooth and continuous surfaces without visible aggregation or deformation. The particles appeared discrete and well-defined, indicating successful stabilization during the formulation process. The size distribution analysis revealed particle diameters predominantly in the range of 50–55 nm, with a mean diameter of approximately 50 nm, consistent with intact viral dimensions of DCV and LJV with particle sizes of 30 nm in diameter [33,34]. The untreated DCV virions (Figure 4b) displayed a markedly different morphology. Low-magnification micrographs revealed a branched, filamentous pattern in which bright, globular structures appeared along interconnected networks. Higher-magnification images indicated that these aggregates consisted of clustered viral particles and irregularly shaped assemblies. The individual particle contours were less distinct compared to the formulated sample, suggesting partial fusion or aggregation on the substrate. Size distribution analysis yielded a distribution centered around 45 nm, but with increased polydispersity relative to LJV. The untreated LJV virions (Figure 4c) presented sparsely distributed, isolated particles on the substrate. Higher-magnification micrographs confirmed the presence of small, spherical particles with well-defined edges and a compact morphology. The insert highlights one particle, showing a consistent, smooth surface texture with visible features of the surface proteins. The measured particle diameters ranged between 35 nm and 47 nm, with an average of approximately 37 nm, indicating a uniform particle population.

Collectively, the SEM observations confirm clear morphological differences between the formulated and untreated virus preparations, as well as between naked DCV and naked LJV. The accompanying histograms summarize the particle size distributions obtained from multiple fields of view. Consistently, the figures provided in the Supplementary Information show similar structural features for encapsulated LJV and DCV. Moreover, light microscopy images of the carrier materials without virus reveal large aggregates that form a continuous surface film upon drying as expected for a cross-linked polymer.

### 3.4. Encapsulation Efficiency

**Figure 5:**
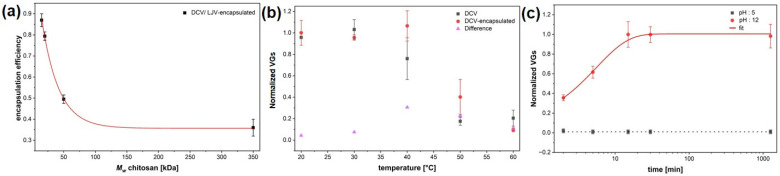
(**a**) Encapsulation efficiency shown for different molecular weights of chitosan (350 kDa; 50 kDa; 20 kDa; 15 kDa). (**b**). Thermal stability of tech. Virus and encapsulated Drosophila C Virus. (**c**) qPCR release kinetics of viral genomes under acidic (pH 5) and alkaline (pH 12) conditions. The encapsulation efficiencies reported in (**a**) correspond to the smallest particle size (Figure 3d).

The encapsulation efficiency of different DCV formulations was quantified by qPCR analysis and revealed clear variations depending on the polymer type and formulation approach (Figure 5a). Although DCV (Dicistroviridae) and LJV (Iflaviridae) are taxonomically distinct, their nearly identical physicochemical properties—including virion size, non-enveloped icosahedral structure, surface charge, and picorna-like capsid architecture—govern their interactions with chitosan–TPP matrices in an analogous manner. These shared characteristics are the primary determinants of encapsulation efficiency, release behavior, and thermal stability, making DCV a suitable and predictive model for extrapolating formulation performance to LJV. Among the chitosan-based formulations, efficiency increased progressively from the high molecular weight (HMW) chitosan to the ultra-low molecular weight (ULMW) chitosan, reaching values close to 87% ± 5%.

The encapsulation efficiency observed in Figure 5a corresponds to the smallest particle size sample of Figure 3d, indicating that more intense mixing conditions favor both finer particle formation and improved encapsulation performance. Note that the EE represents the total amount of detected RNA, regardless of the viral functionality.

The thermal stability of DCV and its chitosan–TPP encapsulated counterpart was evaluated by quantifying the normalized viral genome equivalents (VGEs) following incubation at increasing temperatures (Figure 5b). Non-formulated DCV exhibited a pronounced decrease in detectable VGEs with increasing temperatures, indicating progressive thermal degradation of the viral capsid above 30 °C. In contrast, the encapsulated DCV maintained nearly constant VGE values up to 40 °C, demonstrating a substantial improvement in temperature resistance conferred by the chitosan coating. At 50 °C, encapsulated samples still retained more than 40% of the initial viral signal, whereas unformulated DCV dropped below 20%. The differential profile (ΔVGE) between coated and uncoated samples clearly reflects the protective effect of the formulation, likely resulting from the thermal shielding and structural stabilization of virions within the chitosan–TPP matrix. These findings confirm that the encapsulation markedly enhances thermal robustness, an essential requirement for field applicability of virus-based biocontrol agents.

Release kinetics were studied at acidic (pH 5) and alkaline (pH 12) conditions (Figure 5c), which is important to simulate the different pH conditions in the interior midgut (pH 7), the acidic region (pH 2) and the posterior midgut (pH 12), where the release should occur. Whereas acidic conditions resulted in negligible release, alkaline conditions induced a rapid increase of viral genomes, reaching a plateau after approximately 30 min with a single exponential trend. The time constant for the release is 336 s ± 42 s, as evaluated by an exponential fit.

### 3.5. Biological Efficacy

**Figure 6:**
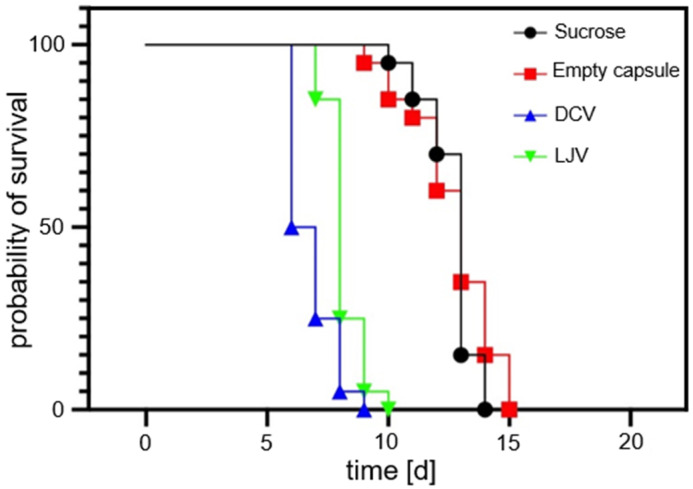
Survival curves of flies treated with Drosophila C Virus and La Jolla Virus compared to sucrose-only and empty formulation control samples. Both viral treatments induced a strong reduction in survival, with a faster onset of mortality seen for Drosophila C Virus. Note that the empty formulation itself did not harm *Drosophila suzukii*.

The survival of *SWD* adults following exposure to the formulated viral treatments was monitored over a period of 20 days. The survival of SWD adults for untreated virions has previously been shown by Linscheid et al. (2022) [36]. The survival analysis results are shown in Figure 6. These results confirm the biocompatibility of the chitosan–TPP capsule formulation. The survival curves of flies treated with the empty formulation were statistically indistinguishable from the sucrose control group (LT_50_ = 13 days; Log-Rank *p* = 0.3655), establishing the matrix’s suitability as a non-toxic carrier for *SWD*.

In contrast, oral administration of both DCV and LJV resulted in a highly significant reduction in lifespan compared to the sucrose control (*p* < 0.0001 for both), providing the first experimental evidence for the oral virulence of these viruses against *SWD*. Kinetic analysis determined the median lethal time (LT_50_ to be 6.5 days for DCV and 8.0 days for LJV). The log-rank Hazard Ratios (HRs) indicated a significant 4-fold increase in the risk of death for the DCV (HR = 4.323) and LJV (HR = 4.058) groups compared to the sucrose control. Although DCV exhibited faster killing kinetics (lower LT_50_), the dose–response analysis suggests LJV is intrinsically more potent (LC_50_ LJV < LC_50_ DCV). In summary, both formulated viruses demonstrated potent pathogenicity, positioning them as promising candidates for a novel viral biopesticide, especially when coupled with the biocompatible chitosan encapsulation strategy.

These findings are consistent with prior work by Linscheid et al. (2022) [36], who demonstrated that oral administration of non-formulated LJV to adult *SWD* significantly reduced survival and also impaired larval development during pupation. Similarly, studies by Lee & Vilcinskas (2017) [37] reported DCV isolated from wild *SWD* populations and highlighted its potential as a biological control agent via injection-based assays. However, these earlier studies noted limited success of oral delivery of DCV in achieving high mortality under natural infection conditions.

Nevertheless, it is noteworthy that the formulated viruses in our experiments caused slower mortality than the rapid injection-based effects reported in the literature, an expected trade-off given the oral exposure route and the requirement for environmentally realistic delivery.

## 4. Discussion

The present study demonstrates the successful formulation of DCV and LJV using a chitosan–TPP system that provides both physicochemical stability and biological efficacy against *SWD*. The results highlight that chitosan-based encapsulation is a promising strategy to protect virions from abiotic stress (acidic pH) while ensuring their controlled release under biologically relevant pH conditions in the posterior midgut (pH 10).

The physicochemical characterization confirmed that electrostatic complexation between chitosan and TPP effectively encapsulated both viruses, as indicated by the increased hydrodynamic diameters (Figure 3a,b) and the shift in autocorrelation functions. The ζ-potential measurements (Figure 3c) revealed that encapsulation is feasible within a narrow pH window where chitosan remains positively charged while the virion’s surface exhibits a slightly negative charge. This charge complementarity is essential for stable formation of nanocapsules (via Coulombic interactions). The SEM images (Figure 4) further support that the chitosan–TPP matrix preserves viral morphology and prevents aggregation. The preservation of particle integrity in the encapsulated samples suggests that the formulation process does not induce significant structural damage to the virions. Encapsulation efficiency (Figure 5a) strongly depended on chitosan molecular weight and formulation parameters (Figure 3d). The observed increase in encapsulation efficiency with decreasing molecular weight can be attributed to the enhanced mobility and flexibility of low-molecular-weight chitosan chains, facilitating closer interactions with the virion’s surface and more compact particle formation. The high encapsulation efficiency (87%) achieved highlights the importance of tailoring polymer composition and process conditions such as stirring speed. The exponential decrease in particle size with increasing homogenization speed further supports that hydrodynamic shear promotes smaller particle formation, leading to more uniform coatings and improved encapsulation of virions. The release kinetics, shown in Figure 5c revealed a strong pH dependence, with negligible release under acidic conditions and rapid genome liberation under alkaline conditions. This behavior aligns well with the physiological environment of the *SWD* gut, where the anterior midgut presents an acidic barrier and the posterior midgut provides more basic conditions conducive to viral release and infection. The measured release time constant (~336 s) indicates that the formulation provides both temporal and spatial control over virus availability, which may enhance infection efficiency while minimizing premature degradation. This pH-responsive release mechanism is consistent with previous findings for chitosan-based delivery systems and underscores the suitability of this polymer for controlled release applications in biocontrol formulations. Although DCV (Dicistroviridae) and LJV (Iflaviridae) belong to different viral families, they exhibit stark morphological and physicochemical similarities in terms of size, structure, and surface charge [33,34] that are decisive for formulation outcome. In the studies presented in Figure 5, DCV served as a model virus. Based on the similarities, we assume that both viruses display comparable behavior with respect to encapsulation efficiency (EE), release characteristics, and thermal stability.

Importantly, the biological assays confirmed that the chitosan–TPP encapsulated DCV and LJV retained their infectivity and induced high mortality in *SWD* (Figure 6). The comparison is drawn between the formulated virions in this study and the unformulated ones, which have previously been described by Linscheid et al. (2022) [36]. The absence of any mortality effect in the empty-formulation controls demonstrates that the carrier matrix itself is biologically inert and does not negatively affect *SWD*. The faster onset of mortality observed for LJV compared to DCV likely reflects intrinsic differences in virulence or infection kinetics between the two viruses, consistent with earlier reports describing LJV as a more rapidly replicating virus in *Drosophila* hosts [29]. The comparable survival dynamics between the empty capsule and sucrose-only controls further validate that the observed effects stem solely from viral infection rather than formulation artifacts.

Taken together, these findings demonstrate that chitosan–TPP formulations effectively stabilize viral particles, protect them under acidic conditions, and enable pH-controlled release under conditions that mimic the target host environment. This system thus represents a significant advance toward the practical application of viral biocontrol agents against *SWD*. Future work should focus on field trials to evaluate environmental persistence, transmission efficiency, and formulation stability under real-world conditions. Additionally, the potential to generalize this approach to other entomopathogenic viruses could broaden its relevance for sustainable pest management strategies.

A major economic bottleneck currently limits the practical application of this approach under field conditions. To elevate the system to a financially viable level, improved purification procedures and substantial upscaling of virus production would be required.

## 5. Conclusions

This study establishes a robust chitosan–TPP encapsulation system for the stabilization and controlled release of DCV and LJV virions. The formulation effectively protects viral particles against acidic and environmental stress while ensuring rapid release under alkaline conditions that mimic the posterior’s midgut environment (infection site in *SWD*). Physicochemical analyses confirmed successful encapsulation and structural preservation of virions, and biological assays demonstrated strong efficacy without formulation-related toxicity. To our knowledge, this represents the first demonstration that cell-culture-derived LJV can infect and lethally affect *SWD* through oral administration, underscoring its potential as a novel viral biocontrol agent when formulated within the chitosan–TPP delivery system. Together, these results highlight chitosan-based formulations as a promising and environmentally sustainable strategy for the delivery of entomopathogenic viruses in pest control applications. Future work should evaluate long-term stability and field performance to facilitate practical implementation in integrated pest management programs.

## Data Availability

The raw data supporting the conclusions of this article will be made available by the authors on request.

## Supplementary Materials

The following supporting information can be downloaded at: https://www.mdpi.com/article/10.3390/insects16121258/s1, Figure S1: SEM Image encapsulated LJV; Figure S2: Optical microscopy image of Chitosan-TPP aggregates; Table S1: DCV Release; Table S2: Encapsulation efficiency data; Table S3: qPCR results of temperature stability; Table S4: Survival analysis (Kaplan-Meier test) comparing the efficacy of the chitosan capsule formulation against the control group; Table S5: Survival analysis comparing the efficacy of the LJV infected group against the control group; Table S6: Survival analysis comparing the efficacy of the LJV infected group against the control group.
