# Supplementary material for: Drosophila C Virus and La Jolla Virus Formulations for Plant Protection Against Spotted-Wing Drosophila
Source: Insects. 2025 Dec 11;16(12):1258. doi: 10.3390/insects16121258 (PMC12734148; doi:10.3390/insects16121258)
Supplement: Supplementary file 1 [file insects-16-01258-s001.zip › insects-4001587-supplementary.pdf]

Drosophila C virus and La Jolla virus formulations for plant protection against *Drosophila suzukii*

Supplementary Information

Monja Jochmann [1,2], Sven Sölmann [1,2], Thorsten Gröb [3], Martin Wortmann [4], Kwang-Zin Lee [5], Michael Wolff [3], Waldemar Keil [1,]* and Anant V. Patel [1,]

1 Faculty of Engineering and Mathematics, Fermentation and Formulation of Biologicals and Chemicals, Hochschule Bielefeld – University of Applied Sciences and Arts, Interaktion 1, 33619 Bielefeld, Germany
2 Department of Technology, Bielefeld University, Universitätsstr. 25, 33615 Bielefeld, Germany
3 Institute of Bioprocess Engineering and Pharmaceutical Technology, University of Applied Sciences Mittelhessen (THM), Giessen, Germany
4 Faculty of Physics, Bielefeld University, Universitätsstr. 25, 33615 Bielefeld, Germany
5 Fraunhofer Institute for Molecular Biology and Applied Ecology, Branch of Bioresources, Ohlebergsweg 12, D-35392 Giessen, Germany

* Correspondence: waldemar.keil@hsbi.de

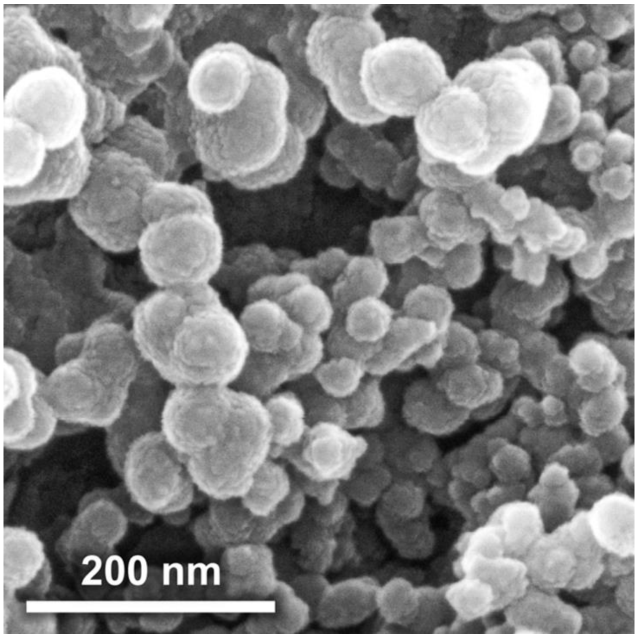

Figure S1: SEM Image encapsulated LJV.

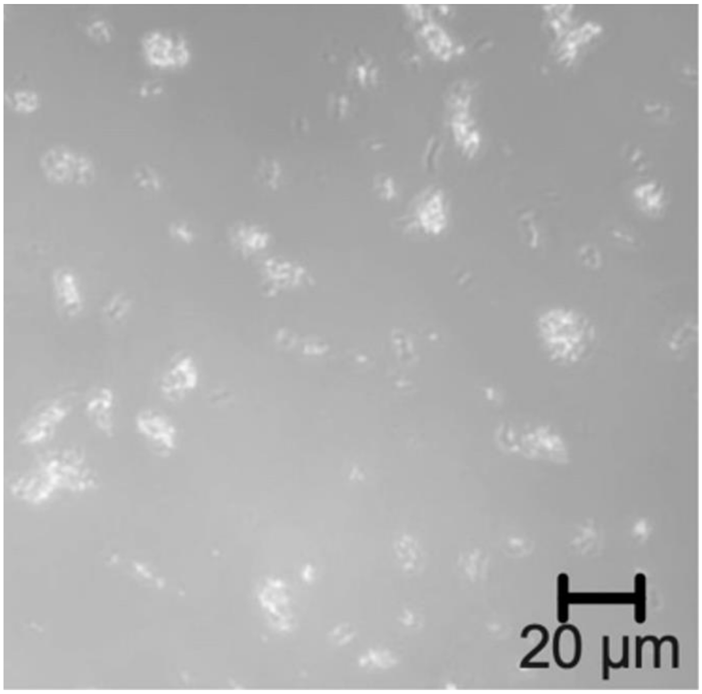

Figure S2: Optical microscopy image of Chitosan-TPP aggregates.

Table S1: DCV Release

	pH5 Pellet (GE/ml)	Supernatant (GE/ml)					
Time (min)		0,00	2	5	15	30	1260
Rep 1	1,83	0,00	1,59	1,20	4,80	4,93	4,57
Rep 2	7,44	0,00	1,58	3,77	4,64	3,43	4,74
Rep 3	4,64	0,00	1,77	3,59	4,48	5,54	4,37
Mean	4,64	0,00	1,65	2,86	4,64	4,63	4,56
SD	2,81	0,00	0,09	1,17	0,13	0,88	0,15

Table S2: Encapsulation efficiency data

	Mean Pellet ($\cdot 10^6$ GE/ml)	Mean Supernatant ($\cdot 10^6$ GE/ml)	Pellet (%)
HMW	9,031	3,251	36
LMW	6,105	6,23	49,5
VLMW	4,176	1,082	79,4
ULMW	52,84	7,875	87

Table S3: qPCR results of temperature stability

Temp		Rep 1 (GE/ml)	Rep 2 (GE/ml)	Rep 3 (GE/ml)	Mean (GE/ml)	SD (GE/ml)
20 ˚C	Neat Virus	1,89E+09	1,95E+09	1,91E+09	1,92E+09	2,49E+07
	Enc. Virus	4,81E+08	5,91E+08	6,40E+08	5,71E+08	6,65E+07
30 ˚C	Neat Virus	2,31E+09	2,01E+09	1,87E+09	2,06E+09	1,84E+08
	Enc. Virus	5,31E+08	5,60E+08	5,92E+08	5,46E+08	1,45E+07
40 ˚C	Neat Virus	1,01E+09	1,60E+09	1,95E+09	1,52E+09	3,88E+08
	Enc. Virus	5,01E+08	6,27E+08	6,95E+08	6,08E+08	8,04E+07
50 ˚C	Neat Virus	3,71E+08	4,31E+08	2,51E+08	3,51E+08	7,48E+07
	Enc. Virus	1,21E+08	3,50E+08	2,17E+08	2,29E+08	9,39E+07
60 ˚C	Neat Virus	3,20E+08	2,90E+08	6,20E+08	4,10E+08	1,49E+08
	Enc. Virus	5,90E+07	6,12E+07	4,10E+07	5,37E+07	9,05E+06

Table S4: Survival analysis (Kaplan-Meier test) comparing the efficacy of the chitosan capsule formulation against the control group

**Table Analyzed**	**Survival 1**	
**Logrank (Mantel-Cox) test**		
**Chi square**	0,8188	
**df**	1	
**P value**	0,3655	
**P value summary**	ns	
**Are the survival curves sig different?**	No	
**Gehan-Breslow-Wilcoxon test**		
**Chi square**	0,1234	
**df**	1	
**P value**	0,7254	
**P value summary**	ns	
**Are the survival curves sig different?**	No	
**Median survival**		

**Control**	13,0000	
**Chit Capsule**	13,0000	
**Ratio (and its reciprocal)**	1,000	1,000
**95% CI of ratio**	0,5380 to 1,859	0,5380 to 1,859
**Hazard Ratio (Mantel-Haenszel)**	A/B	B/A
**Ratio (and its reciprocal)**	1,489	0,6716
**95% CI of ratio**	0,6286 to 3,527	0,2835 to 1,591
**Hazard Ratio (logrank)**	A/B	B/A
**Ratio (and its reciprocal)**	1,229	0,8135
**95% CI of ratio**	0,6592 to 2,292	0,4363 to 1,517

Table S5: Survival analysis comparing the efficacy of the LJV infected group against the control group.

**Table Analyzed**	**Data 1**	
**Logrank (Mantel-Cox) test**		
**Chi square**	42,66	
**df**	1	
**P value**	<0,0001	
**P value summary**	****	
**Are the survival curves sig different?**	Yes	
**Gehan-Breslow-Wilcoxon test**		
**Chi square**	36,52	
**df**	1	
**P value**	<0,0001	
**P value summary**	****	
**Are the survival curves sig different?**	Yes	
**Median survival**		
**Control**	13,0000	
**LJV**	8,0000	

**Ratio (and its reciprocal)**	1,625	0,6154
**95% CI of ratio**	0,8743 to 3,020	0,3311 to 1,144
**Hazard Ratio (Mantel-Haenszel)**	A/D	D/A
**Ratio (and its reciprocal)**	0,02937	34,05
**95% CI of ratio**	0,01019 to 0,08466	11,81 to 98,16
**Hazard Ratio (logrank)**	A/D	D/A
**Ratio (and its reciprocal)**	0,2464	4,058
**95% CI of ratio**	0,1132 to 0,5366	1,864 to 8,835

Table S6: Survival analysis comparing the efficacy of the LJV infected group against the control group.

**Table Analyzed**	**Data 1**	
**Logrank (Mantel-Cox) test**		
**Chi square**	44,72	
**df**	1	
**P value**	<0,0001	
**P value summary**	****	
**Are the survival curves sig different?**	Yes	
**Gehan-Breslow-Wilcoxon test**		
**Chi square**	37,13	
**df**	1	
**P value**	<0,0001	
**P value summary**	****	
**Are the survival curves sig different?**	Yes	
**Median survival**		
**Control**	13,0000	
**DCV**	6,5000	
**Ratio (and its reciprocal)**	2,000	0,5000

**95% CI of ratio**	1,076 to 3,717	0,2690 to 0,9293
**Hazard Ratio (Mantel-Haenszel)**	A/C	C/A
**Ratio (and its reciprocal)**	0,02784	35,92
**95% CI of ratio**	0,009744 to 0,07953	12,57 to 102,6
**Hazard Ratio (logrank)**	A/C	C/A
**Ratio (and its reciprocal)**	0,2313	4,323
**95% CI of ratio**	0,1046 to 0,5114	1,955 to 9,558

List of abbreviations

Abbreviation	Definition
Ds	Drosophila suzukii
DCV	Drosophila C virus
LJV	La Jolla virus
CrPV	Cricket Paralysis virus
FHV	Flock House virus
DAV	Drosophila A virus
TPP	Tripolyphosphate
VGE	Viable Genome Equivalents
S2 cells	Schneider 2 cells
MOI	Multiplicity of Infection
TOH	Time of Harvest
RT-qPCR	Reverse Transcription Quantitative Polymerase Chain Reaction
DLS	Dynamic Light Scattering

**PDI**	**Polydispersity Index**
**ELS**	**Electrophoretic Light Scattering**
**EE**	**Encapsulation Efficiency**
**HMW**	**High Molecular Weight**
**LMW**	**Low Molecular Weight**
**VLMW**	**Very Low Molecular Weight**
**ULMW**	**Ultra Low Molecular Weight**
**SEM**	**Scanning Electron Microscope**
**UV**	**Ultraviolet**
**LT50**	**Median Lethal Time**
**HR**	**Hazard Ratio**
**CI**	**Confidence Interval**
